# Predictive markers for head and neck cancer treatment response: T1rho imaging in nasopharyngeal carcinoma

**DOI:** 10.1007/s00330-024-10948-5

**Published:** 2024-08-27

**Authors:** Qi Yong H. Ai, Ann D. King, Yip Man Tsang, Ziqiang Yu, Kaijing Mao, Frankie K. F. Mo, Lun M. Wong, Ho Sang Leung, Tiffany Y. So, Edwin P. Hui, Brigette B. Y. Ma, Weitian Chen

**Affiliations:** 1 Department of Health Technology and Informatics, The Hong Kong Polytechnic University, Hong Kong S.A.R., P.R. China; 2 Department of Imaging and Interventional Radiology, The Chinese University of Hong Kong, Prince of Wales Hospital, Hong Kong S.A.R., P.R. China; 3 Department of Clinical Oncology, The Chinese University of Hong Kong, Prince of Wales Hospital, Hong Kong S.A.R., P.R. China; 4 State Key Laboratory of Translational Oncology, The Chinese University of Hong Kong, Hong Kong S.A.R., P.R. China; 5 Li Ka Shing Institute of Health Sciences, The Chinese University of Hong Kong, Prince of Wales Hospital, Hong Kong S.A.R., P.R. China

**Keywords:** Nasopharyngeal carcinoma, Head and neck, T1rho imaging, Diffusion-weighted imaging, Treatment response

## Abstract

**Objectives:**

To investigate the potential of T1rho, a new quantitative imaging sequence for cancer, for pre and early intra-treatment prediction of treatment response in nasopharyngeal carcinoma (NPC) and compare the results with those of diffusion-weighted imaging (DWI).

**Materials and methods:**

T1rho and DWI imaging of primary NPCs were performed pre- and early intra-treatment in 41 prospectively recruited patients. The mean preT1rho, preADC, intraT1rho, intraADC, and % changes in T1rho (ΔT1rho%) and ADC (ΔADC%) were compared between residual and non-residual groups based on biopsy in all patients after chemoradiotherapy (CRT) with (*n* = 29) or without (*n* = 12) induction chemotherapy (IC), and between responders and non-responders to IC in the subgroup who received IC, using Mann–Whitney *U*-test. A *p*-value of < 0.05 indicated statistical significance.

**Results:**

Significant early intra-treatment changes in mean T1rho (*p* = 0.049) and mean ADC (*p* < 0.01) were detected (using paired *t*-test), most showing a decrease in T1rho (63.4%) and an increase in ADC (95.1%). Responders to IC (*n* = 17), compared to non-responders (*n* = 12), showed higher preT1rho (64.0 ms vs 66.5 ms) and a greater decrease in ΔT1rho% (− 7.5% vs 1.3%) (*p* < 0.05). The non-residual group after CRT (*n* = 35), compared to the residual group (*n* = 6), showed higher intraADC (0.96 vs 1.09 × 10^−3^ mm^2^/s) and greater increase in ΔADC% (11.7% vs 27.0%) (*p* = 0.02).

**Conclusion:**

Early intra-treatment changes are detectable on T1rho and show potential to predict tumour shrinkage after IC. T1rho may be complementary to DWI, which, unlike T1rho, did not predict response to IC but did predict non-residual disease after CRT.

**Clinical relevance statement:**

T1rho has the potential to complement DWI in the prediction of treatment response. Unlike DWI, it predicted shrinkage of the primary NPC after IC but not residual disease after CRT.

**Key Points:**

- *Changes in T1rho were detected early during cancer treatment for NPC*.
- *Pre-treatment and early intra-treatment change in T1rho predicted response to IC, but not residual disease after CRT*.
- *T1rho can be used to complement DWI with DWI predicting residual disease after CRT*.

## Background

T1rho imaging is a new MRI research sequence with a wide range of potential clinical applications in the musculoskeletal system, brain, liver, and heart [1–10], but there is a paucity of cancer-related research with only a few reports on cancer of the prostate, head and neck, breast and rectum [11–14].

The biological processes that contribute to the T1rho image of cancers are not clearly understood, but it is known that T1rho values correlate negatively with the macromolecules in the extracellular matrix (ECM), such as collagen and glycosaminoglycans/proteoglycans [7–10, 15–20]. The complex interactions between tumour cells and these ECM macromolecules in the tumour microenvironment play a key role in cancer growth, progression and response to treatment. Collagen, a prominent structural protein associated with increased stromal content, and proteoglycans that regulate many cancer pathways including collagen fibrillogenesis, are both implicated in poor response to treatment [21–23]. T1rho evaluation of ECM macromolecules therefore could provide new imaging biomarkers to predict and assess cancer treatment response. Potentially, low pre-treatment T1rho values (high ECM macromolecules) may indicate poor outcome and early intra-treatment rises in T1rho values (fall in T1rho values) may indicate good treatment response. However, this view is likely to be too simplistic and at this early stage, it is unknown if T1rho has the potential to predict treatment response.

T1rho research is needed into cancers treated by radiotherapy or chemoradiotherapy (CRT). One such cancer is nasopharyngeal carcinoma (NPC). This cancer is a common head and neck cancer in our practice, primary treatment is CRT in advanced or bulky diseases, and poor outcome has been linked to high stromal content [24, 25]. We have shown that T1rho in the head and neck has good test-retest repeatability of T1rho [26] and can distinguish between NPC and benign hyperplasia [13]. Furthermore, it would be advantageous to identify new predictive imaging biomarkers in NPC to help tailor induction chemotherapy (IC) and concurrent chemoradiotherapy (CCRT) regimens and post-treatment imaging strategies to target individuals at higher risk of residual or recurrent disease.

In this prospective preliminary T1rho study, we took the first steps by determining if changes in T1rho values can be detected in the primary NPC early intra-treatment and, if so, could these changes, together with the absolute pre- and intra-treatment T1rho values, predict short-term response. In a subgroup of patients undergoing IC before CCRT, we evaluated T1rho also for prediction of IC response. The results for T1rho were compared with those of diffusion-weighted imaging (DWI) using the apparent diffusion coefficient (ADC). DWI is one of the most promising functional MRI techniques for the treatment response prediction in head and neck cancers, a high pre-treatment ADC and low early intra-treatment rise in ADC being associated with poor outcomes [27]. Moreover, a high pre-treatment ADC has been associated with high stromal content [28]. Both DWI and T1rho are relatively fast sequences without intravenous contrast administration, but early studies suggest that T1rho has an advantage over DWI because it shows less variability and image distortion from B0 field inhomogeneities [29, 30].

## Materials and methods

### Participants

**Fig. 1:**
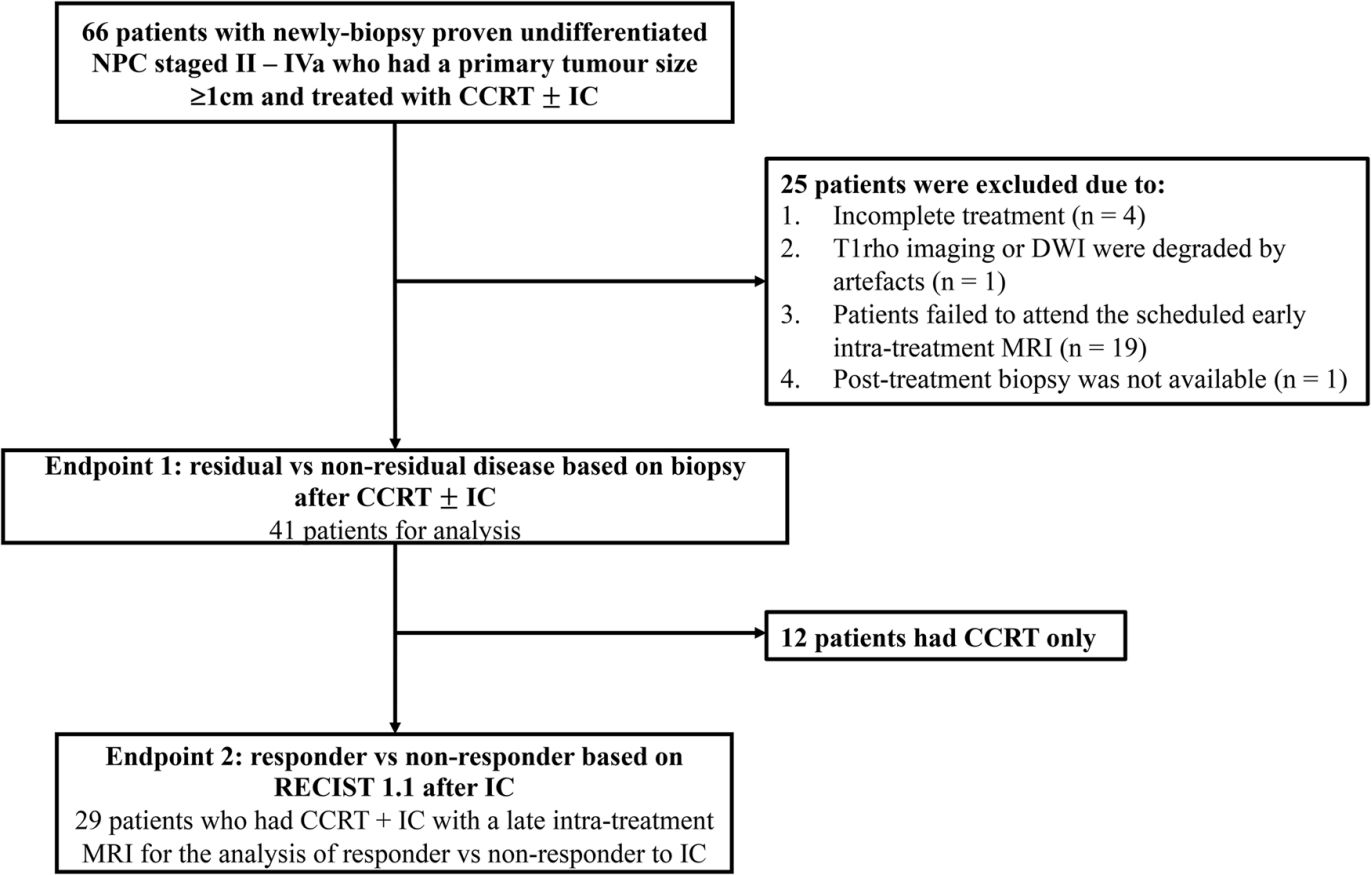
A flowchart of patient selection. NPC, nasopharyngeal carcinoma; CCRT, concurrent chemotherapy; IC, induction chemotherapy; DWI, diffusion-weighted imaging; MRI, magnetic resonance imaging

This prospective study was performed with local institutional board approval. Written informed consent was obtained from each eligible patient. Patient recruitment for the study followed the inclusion criteria: (1) adult patients with newly biopsy-proven undifferentiated NPC; (2) pre-treatment staging MRI performed between June 2020 and October 2022 which showed stage II–IVa NPC; (3) size of primary NPC ≥ 1 cm in the axial plane and (4) treatment included concurrent CCRT with or without IC for curative intent and exclusion criteria: (a) incomplete treatment; (b) images degraded by artefact, (c) patient unable to attend the scheduled early intra-treatment MRI, or (d) post-treatment biopsy was unavailable. A flowchart for patient selection is shown in Fig. 1.

### MRI

MRI was performed on a Philips Achieva TX 3-T scanner (Philips Healthcare) with a body coil for radiofrequency transmission and a 16-channel Philips neurovascular phased-array coil for reception. T1rho and DWI were performed pre-treatment by adding these sequences to our clinical NPC staging protocol in the routine workflow. T1rho and DWI were repeated early intra-treatment at two weeks after the start of chemotherapy, i.e. for patients with CCRT only, the early intra-treatment MRI was performed two weeks after the start of concurrent chemotherapy; for patients with additional IC before CCRT, the early intra-treatment MRI was performed two weeks after the start of IC. The intra-treatment MRI was a short protocol MRI for research that was additional to the clinical workflow. The subgroup of patients undergoing IC also underwent a routine clinical post-IC MRI (without T1rho and DWI) performed for radiotherapy planning before the start of CCRT.

T1rho imaging was performed using an adiabatic continuous wave constant amplitude spin-lock approach [29] followed by single-shot turbo spin-echo acquisition. The spin-lock radiofrequency pulse cluster consisted of a constant amplitude spin-lock radiofrequency pulse sandwiched by an adiabatic half passage (AHP) and a reverse adiabatic half passage (rAHP). Hyperbolic secant pulses were used as the AHP and rAHP, with the B1 amplitude of the AHP and rAHP set equal to that of the spin-lock radiofrequency pulse [29, 30]. In summary, the imaging parameters were: repetition time/echo time, 2500/15 ms; field of view, 230 mm × 216 mm; resolution, 1.2 mm × 1.2 mm; slice thickness, 4 mm; number of slices, 9; sensitivity encoding factor, 2; AHP and rAHP duration, 25 ms; the maximum amplitude of frequency waveform modulation of the AHP and rAHP: 400 Hz; coefficient factor β for AHP and rAHP, 4; frequency of spin-lock, 400 Hz; and spin-lock time, 0 ms, 10 ms, 30 ms, 55 ms, and 90 ms (full details of the pulse sequence are reported previously [31]). The total T1rho imaging scan time was 70–110 s.

DWI was acquired from all patients using the same fat-suppressed, single-shot spin-echo echo-planar imaging sequence. The imaging parameters were repetition time/echo time, 2000/15 ms; field of view, 230 mm × 230 mm; resolution, 1.7 mm × 2.1 mm; slice thickness, 4 mm; number of slices: 9; echo train length, 55; sensitivity encoding factor, 2; number of signals acquired, 4, and six *b*-values (0 s/mm^2^, 200 s/mm^2^, 400 s/mm^2^, 600 s/mm^2^, 800 s/mm^2^ and 1000 s/mm^2^).

Anatomical MRI sequences included at least (1) axial T1-weighted image (T1WI), (2) axial fat-suppressed T2-weighted image (FST2WI), (3) coronal T1WI and (4) contrast-enhanced T1WI with and without fat suppression (contrast-enhanced images were acquired in the routine workflow pre-treatment and post-IC scans).

### Imaging analysis

T1rho images were reconstructed at matrix size 288 × 288. These images were used for T1rho quantification using an in-house Matlab (Mathworks) program. The images were smoothed by a sliding 2 × 2 window throughout the image before quantification. At each pixel, the image intensity was fitted to the relaxation model [13, 26] to calculate the T1rho value, where A and B are two unknown constants. We used a dichotomy method (29) variant to fit the data to this relaxation model to quantify T1rho values. For on-resonance spin-lock, the DC term is positive. This condition was incorporated in the fitting algorithm to improve fitting accuracy. The peak signal-to-noise ratio (PSNR) was calculated to evaluate the goodness of fit [13, 26]. Criteria were set to exclude pixels with obvious errors or possible unreliable fitting results. A pixel was excluded from the final analysis if it had PSNR < 30 or an extreme T1rho value (< 15 ms or > 200 ms).

Olea Sphere (version 3.0; Olea Medical SA) was used for the diffusion post-processing steps by implementing a Bayesian probability-based algorithm using all six *b*-values (0 s/mm^2^, 200 s/mm^2^, 400 s/mm^2^, 600 s/mm^2^, 800 s/mm^2^, and 1000 s/mm^2^) to fit a mono-exponential diffusion model to calculate the conventional ADC.

The primary tumour on the pre- and intra-treatment T1rho maps and ADC maps was contoured manually, excluding any necrotic or cystic areas with reference to the corresponding anatomical images by a doctor and researcher with 8 years of experience in MRI of NPC and were checked by a radiologist with over 25 years of experience in NPC. The contours of primary tumours on the T1rho maps were performed again by the same researcher with a time interval of about 14 days and a radiologist with 3 years of experience in head and neck radiology, respectively, for the evaluation of intra- and inter-observer agreements for the T1rho value. The mean T1rho values of primary tumours obtained from the first set of contours were used for analysis. The mean values of pre- and intra-treatment T1rho (preT1rho and intraT1rho) and ADC (preADC and intraADC), and changes in T1rho and ADC between pre- and intra-treatment scans, which were defined as ΔT1rho% = [(intraT1rho-preT1rho)/preT1rho × 100%], and ΔADC% = [(intraADC-preADC)/ preADC × 100%], were calculated for further analysis.

### Tumour staging

The primary tumour and nodal metastases on the pre-treatment MRI were staged based on the 8th edition of the American Joint Committee on Cancer/Union for International Cancer Control Cancer Staging Manual.

### Treatment

All patients received standardized treatment protocol, i.e. concurrent chemotherapy + intensity modulated radiation therapy with or without IC. For the radiation therapy, the primary tumour and grossly enlarged lymph nodes received 66–70 grey and regions at risk of microscopic spread and the bilateral cervical lymphatics were selectively irradiated to 50–60 grey in 33–35 fractions. The standard concurrent chemotherapy regime was mainly weekly low-dose (40 mg/m^2^) cisplatin for 4–7 cycles. The standard IC regimen was gemcitabine at a dose of 1 g/m^2^ of the body-surface area on days 1 and 8, and cisplatin at a dose of 80 mg/m^2^ on day 1, administered intravenously once every three weeks for 2–3 cycles.

### Endpoints

Residual and non-residual disease after CRT (all patients with CCRT alone or combined with IC)

Patients underwent clinical examination which included endoscopy with biopsy of the primary tumour bed 6–8 weeks after the completion of CRT. Patients with or without histologically positive undifferentiated carcinoma were classified into residual and non-residual NPC groups, respectively.

Responder and non-responder after IC (subgroup of patients with IC)

For the subgroup of patients treated with IC before CCRT, treatment response was evaluated by the percentage change in size of the primary tumour between the pre-treatment MRI and MRI performed at the end of IC, using the RECIST 1.1 criteria [32] to divide patients into responders (complete response (CR) or partial response (PR)) and non-responders (stable disease (SD) or progressive disease (PD)).

### Statistical analysis

Differences in T1rho and ADC values between pre- and early intra-treatment scans were evaluated using the paired student *t*-test. For the analysis of both the residual vs non-residual group and the responder vs non-responder group, the differences of the continuous variables (preT1rho, intraT1rho, ΔT1rho%, preADC, intraADC, ΔADC% and age) and categorical variables (T1–2 vs T3–4, N0–1 vs N2–3 and sex) were compared using Mann–Whitney *U*-test and Chi-square test/Fisher’s exact test, respectively. Receiver-operating characteristic curve analysis and area under the curve (AUC) calculations of significant variables were used to identify the optimal thresholds by maximizing the sensitivity plus specificity. The significance of these optimal thresholds was re-evaluated with the Chi-square test. The sensitivity, specificity and accuracy of the optimal thresholds were calculated and the AUCs were compared using the DeLong test [33]. Intra- and inter-observer agreement for T1rho values were evaluated using the intra-class correlation test, and the intra-class coefficient (ICC) was calculated. Intra- and inter-observer analyses for DWI were not analysed as previous NPC studies have shown the ICCs for ADC of > 0.90 [34, 35]. All of the statistical tests were two-sided, and a *p*-value < 0.05 was considered to indicate a statistically significant difference. Analyses were performed using the statistical analysis software SPSS (version 25.0; IBM) and open-source Python package (Scikit-learn version 1.3.0 in Python version 3.8.0).

## Results

### Participants

Prospectively, 66 eligible patients were recruited for the study. After excluding 25 patients, incomplete treatment (*n* = 4), images degraded by an artefact (*n* = 1), failure to undergo the early intra-treatment MRI (two weeks after the start of chemotherapy) (*n* = 19); and post-treatment biopsy not available (*n* = 1), there were 41 patients for the analysis (Fig. 1). Patient demographics, cancer staging, and treatment are shown in Table 1. The median interval time between the start of chemotherapy and the date of early intra-treatment MRI was 14 days (range 11–19 days). Residual disease on biopsy 6–8 weeks after the completion of CRT was present in 6/41(14.6%) patients and absent in 35/41 (85.4%) patients.

**Table 1:** Patient demographics and cancer staging

	Patients with CCRT (%), *N* = 41	Patients with IC (%), *N* = 29
Age (year)		
Median	56	62
Range	29–77	34–74
Sex		
Male	31 (75.6%)	21 (72.4%)
Female	10 (24.4%)	8 (27.6%)
T category		
T1–2	16 (39.0%)	9 (31.0%)
T3–4	25 (61.0%)	20 (69.0%)
N category		
N0–1	20 (48.8%)	9 (31.0%)
N2–3	21 (51.2%)	20 (69.0%)

*CRT* chemoradiotherapy, *IC* induction chemotherapy

Of the 41 patients, 29 had IC with an MRI performed at the end of IC to evaluate IC response (Fig. 1). Patient demographics, cancer stage, and treatment are shown in Table 1. At the end of IC, responder and non-responder were present in 17/29 (58.6%) (CR: *n* = 2, PR: *n* = 15) patients and 12/29 (41.4%) (SD: *n* = 11, and PD: *n* = 1), respectively.

### Treatment-induced changes in T1rho and ADC values

Differences between pre- and intra-treatment scans were statistically significant for mean T1rho (66.8 ± 5.7 vs 64.9 ± 6.2 × ms, *p* = 0.049) and mean ADC (0.85 ± 0.13 vs 1.06 ± 0.12 × 10^−3^ mm^2^/s, *p* < 0.01). The mean T1rho and ADC increased in 15/41 (36.6%) patients (ΔT1rho% ranged from 0.1% to 17.2%) and 39/41 (95.1%) patients (ΔADC% ranged from 0.8% to 84.9%), respectively, and decreased in 26/41 (63.4%) patients (ΔT1rho% ranged from − 24.1% to − 0.1%) and in 2/41 (4.9%) patients (ΔADC% ranged from − 12.3% to − 3.5%), respectively. Analyses showed ICCs of > 0.90 for pre-treatment and intra-treatment T1rho intra-observer and inter-observer agreements.

### Residual and non-residual groups after CCRT with or without IC (*n* = 41)

**Fig. 2:**
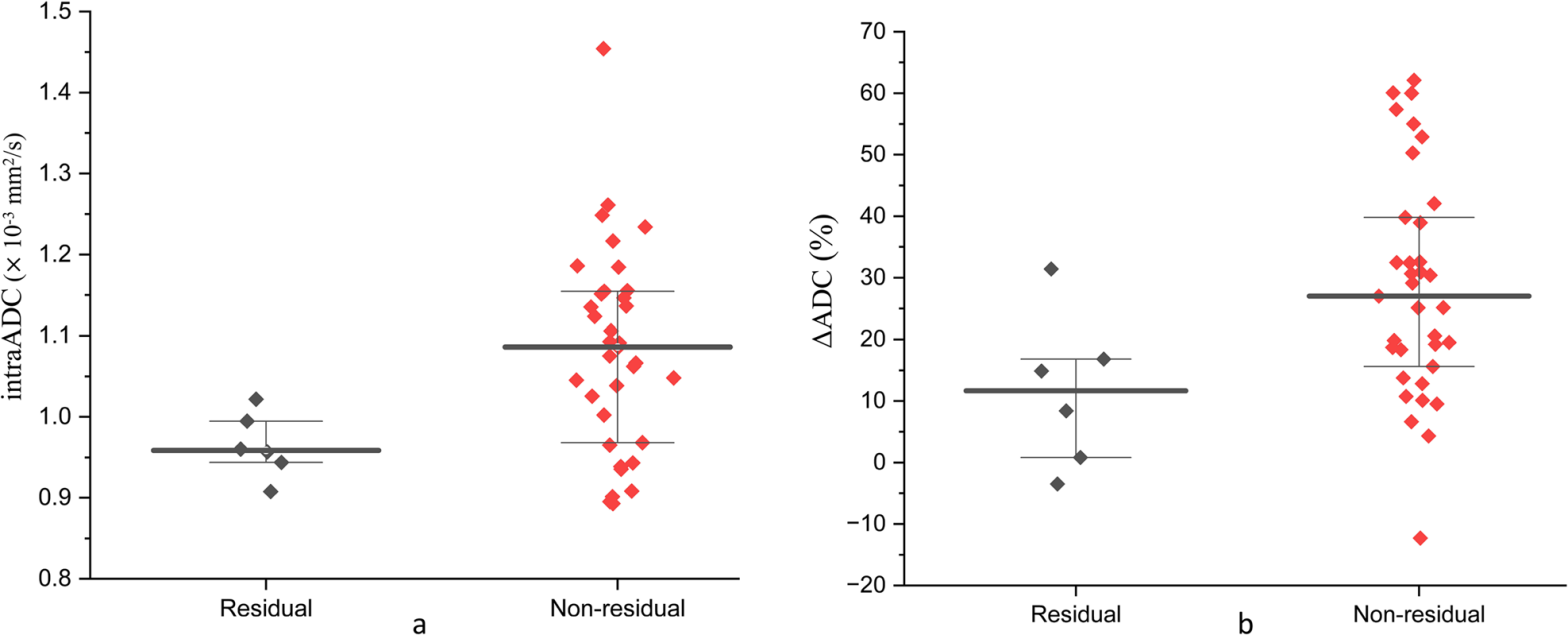
Scattered plots showing intraADC (**a**) and ΔADC% (**b**) in the residual and non-residual NPC based on biopsy after CCRT ± IC. Compared to the residual group, the non-residual group shows a higher intraADC (0.96 vs 1.02 × 10^−3^ mm^2^/s, *p* = 0.02) and greater increase in ΔADC% when compared with (11.7% vs 27%, *p* = 0.02). Bold line indicates the median value, and the upper and lower boundary lines indicate the interquartile range. NPC, nasopharyngeal carcinoma; CCRT, concurrent chemotherapy; IC, induction chemotherapy; ADC, apparent diffusion coefficient

For the residual and non-residual groups, T and N categories, age, gender and the pre- and early intra-treatment T1rho and ADC values (preT1rho, preADC, intraT1rho, intraADC, ΔT1rho% and ΔADC%) are shown in Table 2. There were no statistical differences between the two groups in the T category, N category, age, or gender (*p* = 0.06 to > 0.99), or in any of the T1rho results (*p* = 0.10–0.56) (Table 2). For DWI, there was no difference in the preADC between the two groups (*p* = 0.51), but there was a significantly higher intraADC and greater increase in ΔADC% (*p* = 0.02 and 0.02, respectively) in the non-residual compared to the residual group (Table 2 and Fig. 2). For DWI, the AUCs of intraADC and ΔADC% for the identification of non-residual vs residual groups were 0.80 (95% CI: 0.64–0.91) and 0.79 (95% CI: 0.51–0.93), respectively, with no statistical difference between the two ADC measurements (*p* = 0.96). Performance for the prediction of residual disease using the optimal thresholds of ≤ 1.02 × 10^−3^ mm^2^/s for intraADC and ≤ 16.8% for ΔADC% are shown in Table 3.

**Table 2:** Pre- and early intra-treatment imaging biomarkers, cancer staging, and patient demographics in the residual vs non-residual groups after CCRT ± IC and in the responder and non-responder groups after IC

	Residual and non-residual disease based on biopsy after CCRT ± IC, (*N* = 41)			Responder and non-responder to IC based on RECIST 1.1 after IC, (*N* = 29)		
	Residual disease, (*N* = 6)	Non-residual disease, (*N* = 35)	*p*-value	Responder, (*N* = 17)	Non-responder, (*N* = 12)	*p*-value
PreT1rho (ms)	66.8 (64.0, 70.1)	64.7 (58.7, 67.6)	0.21	66.5 (65.8, 73.9)	64.0 (62.1, 68.2)	**0.03**
IntraT1rho (ms)	64.5 (61.9, 67.6)	60.7 (55.4, 67.0)	0.10	61.9 (60.2, 67.3)	65.3 (62.6, 66.6)	0.17
ΔT1rho%	− 2.6% (− 9.1%, 3.5%)	− 5.9% (− 10.5%, 1.2%)	0.56	− 7.5% (− 12.8%, − 4.1%)	1.3% (− 5.2%, 5.5%)	**0.02**
PreADC (× 10^−3^ mm^2^/s)	0.86 (0.81, 0.95)	0.83 (0.76, 0.92)	0.51	0.83 (0.78, 0.91)	0.89 (0.81, 0.94)	0.28
IntraADC (× 10^−3^ mm^2^/s)	0.96 (0.93, 1.00)	1.09 (0.97, 1.15)	**0.02**	1.00 (0.91, 1.09)	1.03 (0.97, 1.12)	0.30
ΔADC%	11.7% (− 0.2%, 20.5%)	27.0% (15.6%, 39.8%)	**0.02**	25.1% (9.8%, 30.5%)	18.3% (9.0%, 31.3%)	0.98
T category						
T1–2	1 (6.3%)	15 (93.7%)	0.38	7 (77.8%)	2 (22.2%)	0.23
T3–4	5 (20.0%)	20 (80.0%)		10 (50.0%)	10 (50.0%)	
N category						
N0–1	3 (15.0%)	17 (85.0%)	> 0.99	5 (55.6%)	4 (44.4%)	> 0.99
N2–3	3 (14.3%)	18 (85.7%)		12 (60.0%)	8 (40.0%)	
Age (years)	68 (58, 72)	54 (47, 63)	0.06	61 (51, 68)	64 (44, 69)	0.88
Sex						
Male	6 (19.4%)	25 (80.6%)	0.31	12 (57.1%)	9 (42.9%)	> 0.99
Female	0 (0%)	10 (100%)		5 (62.5%)	3 (37.5%)	

Continuous data presents as median (interquartile), Bold indicates statistically significant

*CCRT* concurrent chemoradiotherapy, *IC* induction chemotherapy, *ADC* apparent diffusion coefficient

**Table 3:** Performances of DWI for predicting residual from non-residual group after CCRT ± IC and performances of T1rho imaging for predicting responder from non-responder groups after IC

	Identifying residual from non-residual disease after CCRT ± IC, (*N* = 41)		Identifying responder from non-responder after IC, (*N* = 29)		
Imaging biomarkers	intraADC	ΔADC%	preT1rho	ΔT1rho%	Combination of preT1rho and ΔT1rho%
Thresholds	≤ 1.02 × 10^−3^ mm^2^/s	≤ 16.8%	> 64.5 ms	≤ − 3.4%	preT1rho > 64.5 ms or ΔT1rho% ≤ − 3.4%
AUC	0.80 (0.64–0.91)	0.79 (0.51–0.93)	0.74 (0.55–0.88)	0.76 (0.56–0.89)	0.83 (0.65–0.95)
*p*-value	< 0.01	< 0.01	< 0.01	< 0.01	< 0.01
Sensitivity	100% (54.1–100%)	83.3% (35.9–99.6%)	88.2% (63.6–98.5%)	82.4% (56.6–96.2%)	100% (80.5–100%)
Specificity	71.4% (53.7–85.4%)	74.3% (56.7–87.5%)	66.7% (34.9–90.1%)	75.0% (42.8–94.5%)	66.7% (34.9–90.1%)
PPV	37.5% (26.2–50.3%)	35.7% (22.1–51.9%)	78.9% (62.3–89.5%)	82.4% (63.1–92.7%)	81.0% (65.6–90.4%)
NPV	100% (86.3–100%)	96.3% (81.1–99.4%)	80.0% (50.6–94.0%)	75.0% (50.5–89.8%)	100.0% (63.1–100%)
Accuracy	75.6% (59.7–87.6%)	75.6% (59.7–87.6%)	79.3% (60.3–92.0%)	79.3% (60.3–92.0%)	86.2% (68.3–96.1%)

*CRT* chemoradiotherapy, *IC* induction chemotherapy, *DWI* diffusion-weighted imaging, *ADC* apparent diffusion coefficient, *AUC* area under the curve, *PPV* positive predictive value, *NPV* negative predictive value

### Responder and non-responder groups in subgroup after IC (*n* = 29)

Pre- and early intra-treatment T1rho and ADC values (preT1rho, preADC, intraT1rho, intraADC, ΔT1rho% and ΔADC%), T category, N category, age and gender in responder and non-responder groups are shown in Table 2.

**Fig. 3:**
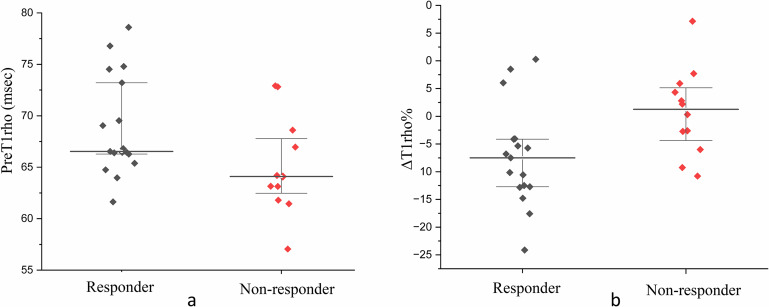
Scattered plots showing the preT1rho (**a**) and ΔT1rho% (**b**) values of the responder and non-responder groups after IC. Compared to the non-responder group, the responder group shows a higher preT1rho value (66.5 vs 64.0 × ms, *p* = 0.03) and lower ΔT1rho% (i.e. greater decrease in the intra-treatment T1rho value) (− 7.5% vs + 1.3%, *p* = 0.02), respectively). The bold line indicates the median value, and the upper and lower boundary lines indicate the interquartile range. IC, induction chemotherapy

For T1rho, the responder group compared to the non-responder group, shows a significantly higher preT1rho and lower ΔT1rho% (i.e. lower percentage increase or a fall in the intra-treatment T1rho value) (*p* = 0.03 and 0.02, respectively) (Fig. 3), but no significant difference in intraT1rho (*p* = 0.17) (Table 2). For DWI, the responder group compared to the non-responder group, shows no significant difference in the preADC, intraADC and ΔADC% (*p* = 0.28–0.98, respectively) (Table 2). Differences in T category, N category, age and gender between the responder and non-responder groups also show no significant difference (*p* = 0.23 to > 0.99) (Table 2).

**Fig. 4:**
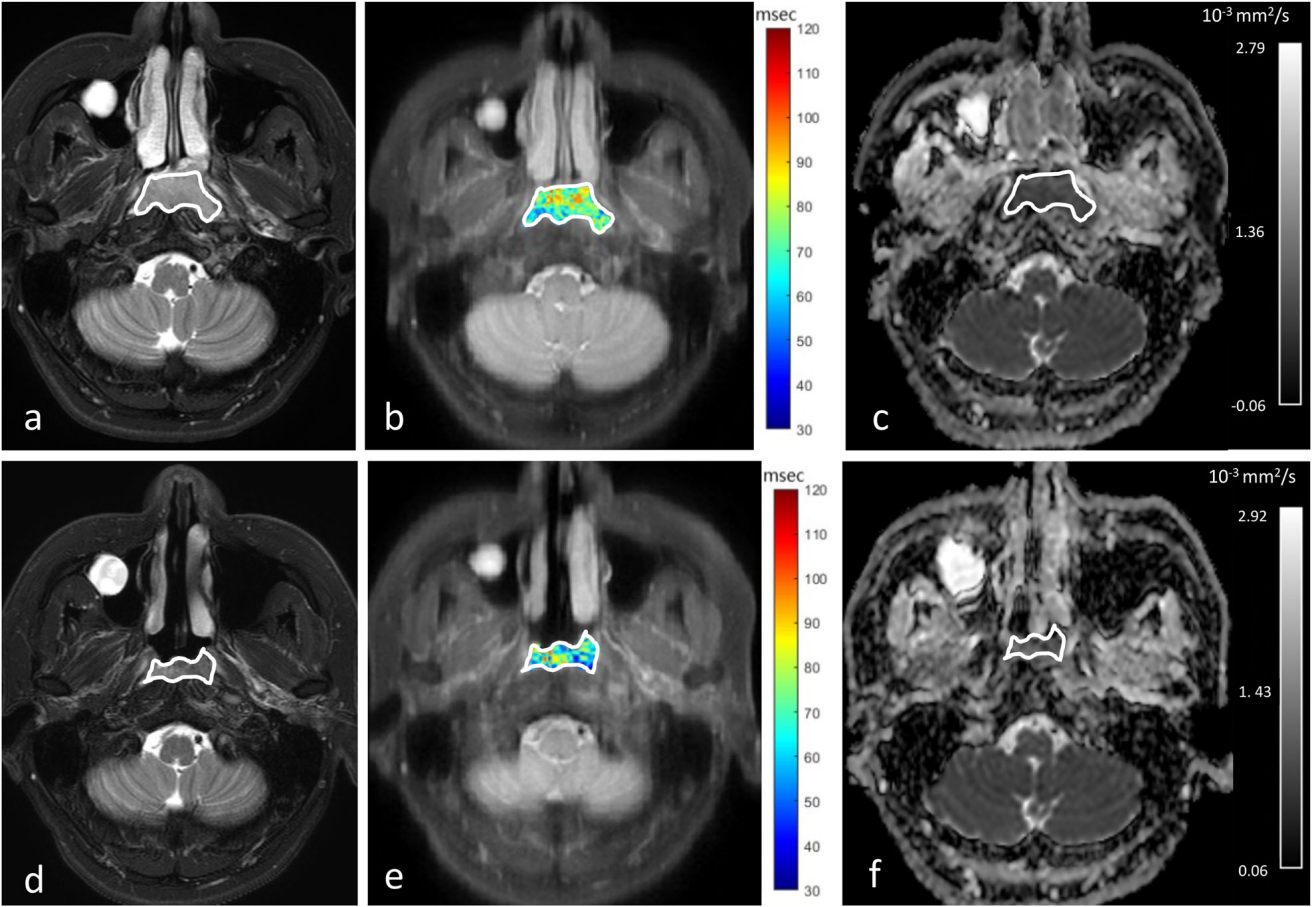
Pre-treatment (**a**–**c**) and early intra-treatment (**d**–**f**) MRIs of a patient with primary NPC that responded to IC and had no residual disease on biopsy after treatment. The axial images comprise T2-weighted fat-suppressed images (**a**, **d**), T1rho maps (**b**, **e**) and ADC maps (**c**, **f**). The Δ% for mean T1rho was − 9.1% and for mean ADC was + 61.2%, respectively. Good response to IC was predicted by a high percentage decrease in the mean T1rho value (i.e. ΔT1rho% of ≤ − 3.4%) and non-residual disease was predicted by a high percentage increase in the mean ADC value (i.e. ΔADC% > + 16.8%). NPC, nasopharyngeal carcinoma; CRT, chemoradiotherapy; IC, induction chemotherapy; ADC, apparent diffusion coefficient; MRI, magnetic resonance imaging

**Fig. 5:**
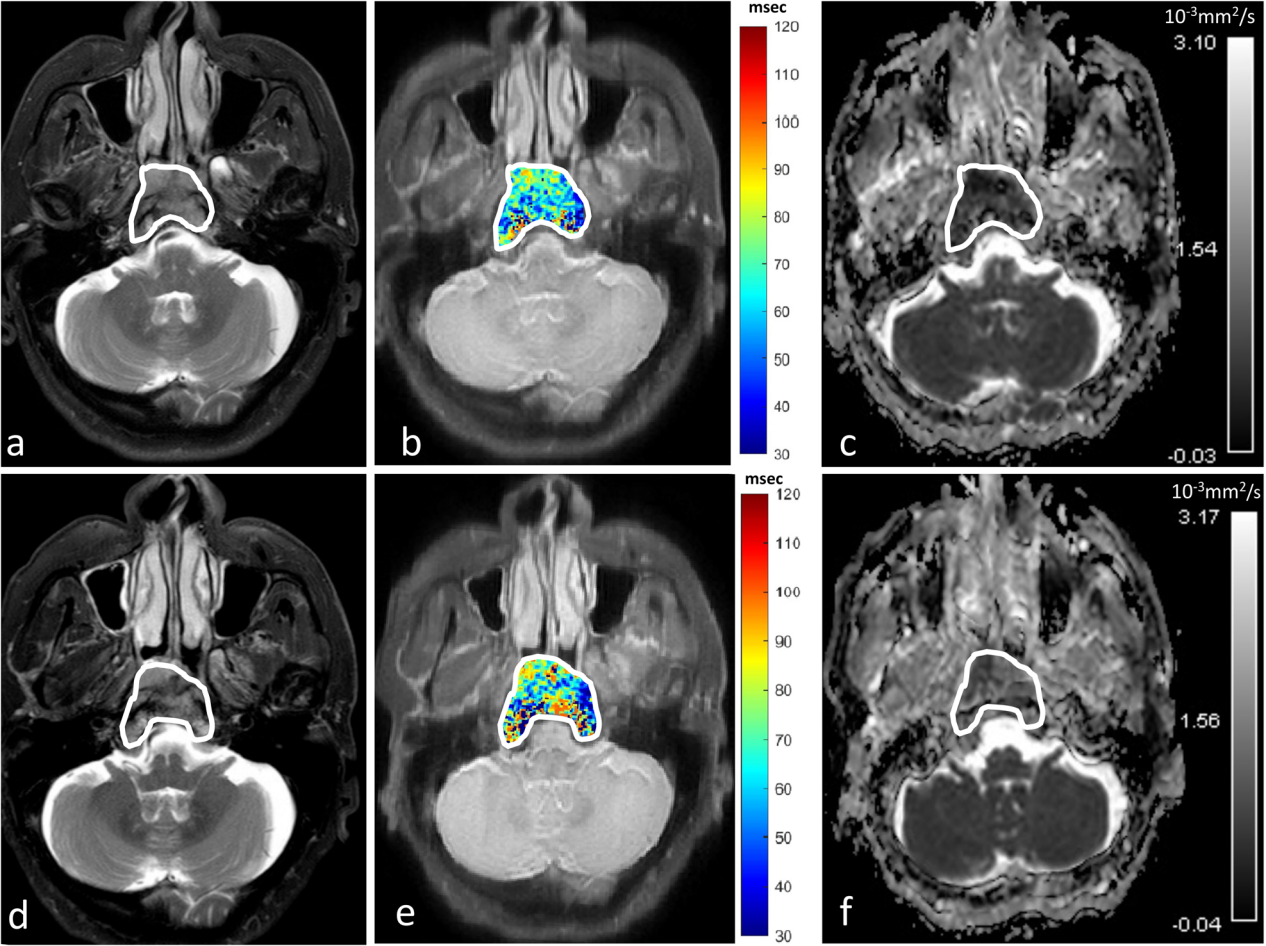
Pre-treatment (**a**–**c**) and early intra-treatment (**d**–**f**) MRIs of a patient with primary NPC that did not respond to IC and showed residual disease on biopsy after treatment. The axial images comprise T2-weighted fat-suppressed images (**a**, **d**), T1rho maps (**b**, **e**) and ADC maps (**c**, **f**). The Δ% for mean T1rho was + 6.0%, and for mean ADC was + 11.3%. Poor response to IC was predicted by a percentage increase in mean T1rho value compared to a decrease for responders (i.e. ΔT1rho% of > − 3.4%) and residual disease was predicted by a lower percentage increase in the mean ADC than expected for responders (i.e. ADC increase was ≤ + 16.8%). NPC, nasopharyngeal carcinoma; CRT, chemoradiotherapy; IC, induction chemotherapy; ADC, apparent diffusion coefficient; MRI, magnetic resonance imaging

The AUCs of preT1rho and ΔT1rho% for the identification of responder and non-responder groups were 0.74 (95% CI: 0.55–0.88) and 0.76 (95% CI: 0.56–0.89), respectively, without a statistical difference between the two AUCs (*p* = 0.91). Performance for the prediction of responder from non-responder using the optimal thresholds of > 64.5 ms for preT1rho and ≤ − 3.4% for ΔT1rho% are shown in Table 3. When using the combination of a ΔT1rho% optimal threshold of ≤ − 3.4% and/or a preT1rho optimal threshold of > 64.5 ms, the sensitivity and NPV increased to 100% and 100%, respectively, for early prediction of responders to IC (Table 3). Figures 4 and 5 show pre- and intra-treatment T1rho and ADC maps of a primary NPC in a patient who responded to IC without residual disease on biopsy after treatment (Fig. 4) and a patient who did not respond to IC and had residual disease on biopsy after treatment (Fig. 5).

## Discussion

This is one of the first cancer studies in any site in the body to evaluate the potential role of quantitative T1rho imaging for the prediction of treatment response [14]. In this study of the primary tumour in NPC, we first evaluated T1rho imaging to predict the presence of residual cancer based on histology taken from the primary tumour bed after the completion of a full course of CRT, with or without IC. Our results showed that the pre-treatment mean T1rho value (preT1rho) was not predictive of residual cancer. We found significant early intra-treatment changes in the mean T1rho value were detectable two weeks after the start of chemotherapy given as IC or CCRT. Both an increase (36.6%) and decrease (63.4%) in mean T1rho values were observed but neither the absolute early intra-treatment measurement (intraT1rho) nor % change (ΔT1rho%), even after considering the expected normal variability, were predictive of residual disease.

The T1rho results from the subgroup analysis of patients who received IC were more encouraging. Responders had higher preT1rho values compared to non-responders to IC, which is in line with our expectations, that a better response could be related to lower collagen and proteoglycans in the cancer ECM. Early intra-treatment changes in T1rho values also predicted response to IC, but contrary to our expectations, some responders to IC showed a lower increase in T1rho values than non-responders, or even a decrease in T1rho values, suggesting responding cancers had less reduction or an increase in macromolecules. One possible explanation is that T1rho imaging detects an increase in the macromolecular content in the ECM from macromolecules released both from tumour cells during apoptosis and from new macromolecules generated in the ECM. It is also possible that other biological mechanisms contribute to the observed changes. The performance of preT1rho and ΔT1rho% for the prediction of IC response were similar, and the best performance was obtained by combining T1rho values at both time points, an optimal threshold for preT1rho of > 64.5 ms and/or ΔT1rho% of ≤ − 3.4%, increased the NPV for the prediction of responders to 100%. For a clinical scenario where the purpose of IC is to shrink the tumour away from vital radiosensitive structures, the accurate prediction of poor tumour shrinkage may aid the decision to curtail the number of IC cycles in those patients experiencing severe IC toxicity. It is also worth noting that the optimal ΔT1rho% threshold of − 3.4% is similar to the reported intrinsic variability of T1rho imaging in the head and neck [26].

The study results show that T1rho may be complementary to DWI because, unlike T1rho imaging, DWI was unable to predict response to IC but could predict response at the end of CCRT. Using the early intra-treatment scan, a lower absolute mean ADC (≤ 1.02 × 10^−^^3^ mm^2^/s) or % increase in mean ADC (ΔADC% ≤ 16.8%) predicted patients with residual cancer on biopsy. Both measurements had a similar performance based on the AUCs, but while the absolute intraADC had the advantage of a high NPV (100%) for predicting residual disease, the ΔADC% measurement may be more reproducible across centres because it is influenced less by differences in technique.

When head and neck cancer cells die the barriers that restrict water molecule movement are broken down and the ADC increases [36]. Our ΔADC% threshold of around 17% for predicting cancers with and without residual disease is encouraging because it is in line with the thresholds for head and neck squamous cell carcinoma (SCC) reported in the literature (14–25%), including a threshold of 15.5% previously reported from our centre [37–42]. These thresholds suggest that residual disease is found in those cancers where the % change is close to, or below, the expected 15% normal variability of DWI [43]. DWI performed pre-treatment was unable to predict residual disease adding to the conflicting results in the literature based on short-term outcomes in NPC [44] and SCC [45].

This study had limitations. First, the prediction of short-term response may not necessarily translate into a prediction of long-term response. Whether or not T1rho imaging can predict long-term treatment response in NPC will require further analysis in the future when the data has matured. Second, the baseline variability of T1rho imaging was chosen based on the reports in the literature of non-parotid regions (the latter may have greater expected variability due to secretory function), but T1rho imaging is still a new technique in cancer imaging and reports on the expected variability are limited. Third, this study did not consider the influence of the MRI scanner on T1rho values, although a previous study in the musculoskeletal system has shown good reproducibility in T1rho values across scanners [46]. Fourth, the study was restricted to only one intra-treatment time point, and it is unknown if this is the optimal time for early T1rho assessment. Fifth, the sample size was small, but the results are still encouraging as the sample was large enough to corroborate the DWI results in the literature.

## Conclusion

T1rho imaging is a new functional MRI technique for cancer imaging, and this is one of the first cancer studies to show that T1rho imaging has the potential to predict treatment response. Early intra-treatment we observed significant changes in the mean T1rho values of the primary tumour in the nasopharynx. These preliminary results suggest a high mean T1rho pre-treatment and a greater % decrease in mean T1rho two weeks after starting IC, predicting better shrinkage of the primary tumour by the end of IC. Results also suggest T1rho imaging and DWI may be complementary, T1rho predicted response to IC while DWI predicted response after CCRT. For DWI, the % increase in mean ADC of > 16.8% in cancers without residual disease is similar to the thresholds reported in the literature for head and neck SCC. Neither technique performed pre-treatment was able to predict residual disease.

Finally, the biological processes in cancers that contribute to the T1rho image are not clearly understood but results from this very early cancer study show that T1rho imaging warrants further investigation in predicting cancer response.

## Abbreviations

ADC: Apparent diffusion coefficient
AHP: Adiabatic half passage
AUC: Area under the curve
CCRT: Concurrent chemoradiotherapy
CR: Complete response
CRT: Chemoradiotherapy
DWI: Diffusion-weighted imaging
IC: Induction chemotherapy
MRI: Magnetic resonance imaging
NPC: Nasopharyngeal carcinoma
PD: Progressive disease
PR: Partial response
SCC: Squamous cell carcinoma
SD: Stable disease
